# Biomarkers of AIT: Models of prediction of efficacy 

**DOI:** 10.5414/ALX02333E

**Published:** 2022-11-21

**Authors:** Tiak Ju Tan, María I. Delgado-Dolset, María M. Escribese, Domingo Barber, Janice A. Layhadi, Mohamed H. Shamji

**Affiliations:** 1 Immunomodulation and Tolerance Group, Department of National Heart and Lung Institute, Imperial College London, London, UK, and; 2 Institute of Applied Molecular Medicine (IMMA), Department of Basic Medical Sciences, Facultad de Medicina, Universidad San Pablo-CEU, CEU Universities, Urbanización Montepríncipe, Boadilla del Monte, Madrid, Spain; *Authors with equal contribution.

**Keywords:** allergen immunotherapy, biomarkers, T cells, metabolomics

## Abstract

Allergic rhinitis is an IgE-mediated inflammation that remains a clinical challenge, affecting 40% of the UK population with a wide range of severity from nasal discomfort to life-threatening anaphylaxis. It can be managed by pharmacotherapeutics and in selected patients by allergen immunotherapy (AIT), which provides long-term clinical efficacy, especially during peak allergy season. However, there are no definitive biomarkers for AIT efficacy. Here, we aim to summarize the key adaptive, innate, humoral, and metabolic advances in biomarker identification in response to AIT. Mechanisms of efficacy consist of an immune deviation towards T_H_1-secreting IFN-γ, as well as an induction of IL10^+^ cT_FR_ and T_REG_ have been observed. T_H_2 cells undergo exhaustion after AIT due to chronic allergen exposure and correlates with the exhaustion markers PD-1, CTLA-4, TIGIT, and LAG3. IL10^+^ DC_REG_ expressing C1Q and STAB are induced. KLRG1^+^ IL10^+^ ILC2 were shown to be induced in AIT in correlation with efficacy. B_REG_ cells secreting IL-10, IL-35, and TGF-β are induced. Blocking antibodies IgG, IgA, and IgG4 are increased during AIT; whereas inflammatory metabolites, such as eicosanoids, are reduced. There are multiple promising biomarkers for AIT currently being evaluated. A panomic approach is essential to better understand cellular, molecular mechanisms and their correlation with clinical outcomes. Identification of predictive biomarkers of AIT efficacy will hugely impact current practice allowing physicians to select eligible patients that are likely to respond to treatment as well as improve patients’ compliance to complete the course of treatment.

## Introduction 

Allergic rhinitis (AR) is an IgE-mediated inflammation of the nasal mucosa triggered by aeroallergens. Symptoms involve rhinorrhea, nasal obstruction, and epiphora [1]. Seasonal AR (SAR) is identified due to onset of symptoms in conjunction with seasonal pollen production, peaking during spring, summer, or fall months [2]. SAR remains a major clinical challenge affecting 10 – 30% of the worldwide population, and generally results in a deteriorated quality of life [3, 4]. Apart from pharmacotherapy, allergen immunotherapy (AIT) remains a key strategy for long-term resolution of symptoms, though long-term clinical benefit is only achieved following treatment of 3 years or longer [5]. For this reason, AIT poses a lot of challenges economically due to the high cost and personally to the patients due to long-term the clinical regimen required to achieve sustained response, resulting in low patient compliance. Biomarkers that allow prediction of AIT efficacy is a huge unmet need in clinical practice and will allow physicians to select eligible patients that are likely to respond to treatment as well as improve patients’ compliance to complete the course of treatment. However, there is currently a lack of validated biomarkers to predict efficacy of AIT. The aim of this review is to outline the key developments in the cellular and metabolite biomarkers as indicative models of effective therapy. 

## Allergic rhinitis 

**Figure 1.**
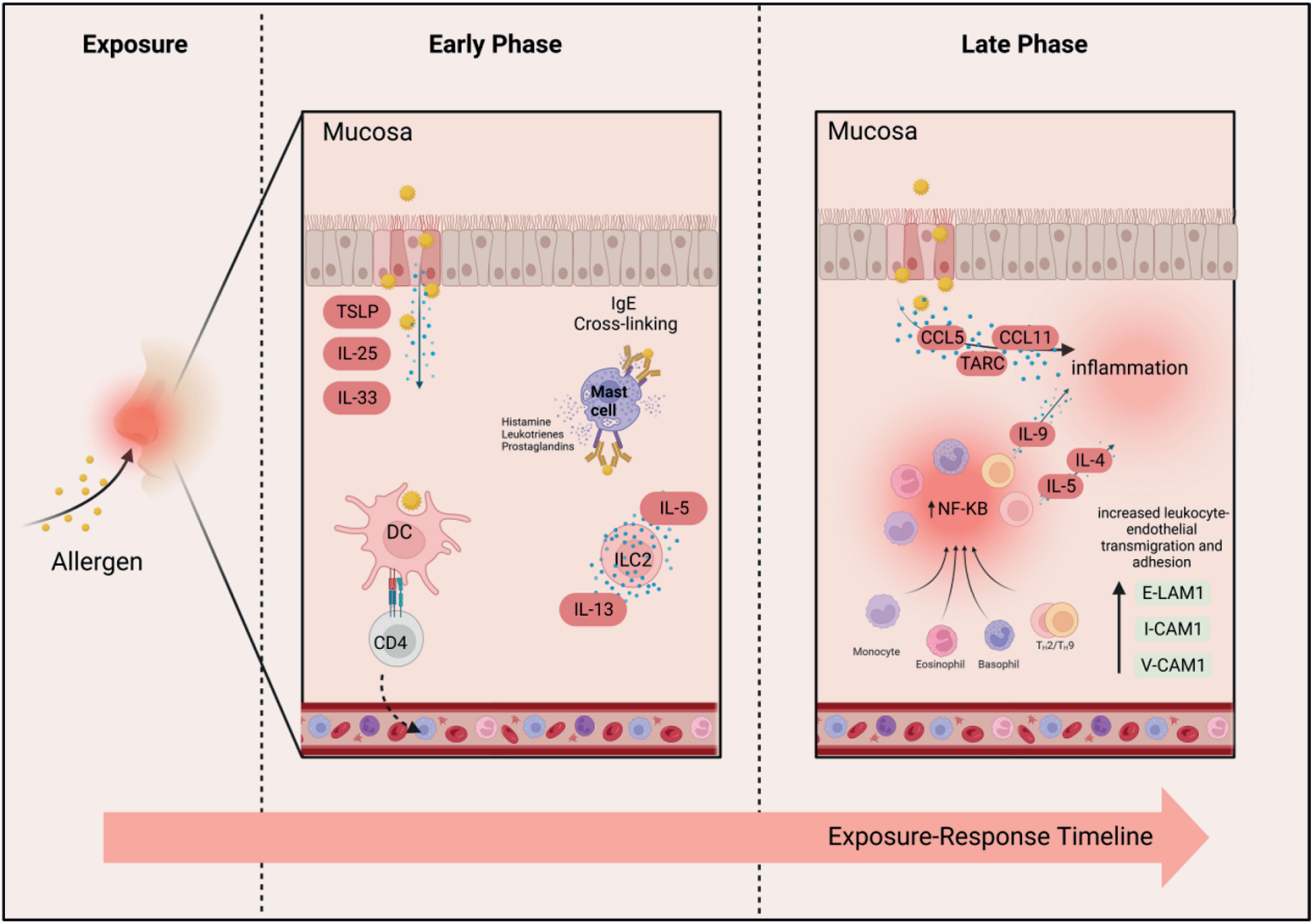
Phases of immune responses after allergen exposure. Early responses in sensitized individuals involve the crosslinking of FcεRI-bound IgE on the surface of mast cells and innate responses, resulting in the release of inflammatory mediators. Late-phase responses involve the infiltration of leukocytes and a general pro-inflammatory state in the local tissue. Created with www.biorender.com.

The early phase of allergic responses in sensitized individuals occurs within minutes and lasts for 3 hours. Offending allergens cross-link IgE on the surface of basophils and mast cells, resulting in the degranulation and release of pro-inflammatory mediators (histamines, leukotrienes, and prostaglandins) [6]. In the late allergic phase, typically within 4 – 12 hours, eosinophils, basophils, T cells, and monocytes infiltrate the airway mucosa, resulting in tissue edema and persistent congestion. Cytokines released by mast cells, such as tumor necrosis factor (TNF), promote pro-inflammatory signaling pathways such as nuclear factor-kappa B (NF-kB) activation and endothelial-leukocyte adhesion molecules such as E-selectin, intracellular adhesion molecule-1 (ICAM-1), and vascular cell adhesion molecule-1 (VCAM-1) [7, 8, 9]. Interleukin (IL)-4 and IL-5, secreted by T_H_2 cells, induce eosinophil recruitment and differentiation, whilst IL-9, secreted by T_H_9 cells, promotes mast cell production and localization [9, 10]. The epithelial barrier also produces RANTES (CCL-5), eotaxin, and thymus- and activation-regulated chemokine (TARC/CCL-17), promoting the tissue localization of granulocytes and T cells [11]. These sustained inflammatory reactions are hallmarks of a late-phase response (Figure 1). Avoiding allergens ablates symptoms, but it is not always feasible. Allergen exposure can be avoided by limiting outdoor interaction during peak pollen seasons. However, a multi-pronged approach involving use of non-sedative second generation oral antihistamines and intra-nasal corticosteroids are more reliable for mild to moderate symptoms [12]. Although widely used, pharmacotherapy provides only temporary relief, while training adaptive immune responses provides superior long-term relief for SAR. Moreover, there is a certain population of individuals who are non-responsive to even high doses of pharmacotherapy. AIT aims to shift the inflammatory profile towards a long-lasting tolerance by inducing key regulatory cells within the immune system. 

## Allergen immunotherapy 

AIT was first performed using grass pollen extracts in patients with SAR before pollen season [13]. It involves the repeated subcutaneous (SCIT) or sublingual (SLIT) administration of high doses of allergens over 3 – 5 years [14]. Both confer long-term clinical benefit and tolerance even after end of treatment. Although AIT is the only disease-modifying treatment for IgE-mediated allergies, there are some limitations, such as associated side effects, poor compliance, and lack of efficacy in non-responders. New approaches to AIT with the aim to enhance safety whilst maintaining or increasing efficacy are currently being researched. A correlation between AIT efficacy and the induction of allergen-neutralizing antibodies as a key biomarker in tolerance induction has been described [15]. The mechanism of action involves the capturing of allergens on mucosal surfaces, inhibiting high-affinity IgE-receptor (FcεRI) activation, preventing low-affinity IgE-receptor (FcεRII/CD23)-mediated antigen presentation, and inhibiting allergic inflammation. On the cellular side, tolerance involves multiple phases and the complex interplay of the innate and adaptive compartments of the immune system, inclusive of effector T cells, dendritic cells (DCs), innate lymphoid cells (ILCs) and B cells. 

## T-cell biomarkers indicating effective AIT 

**Figure 2.**
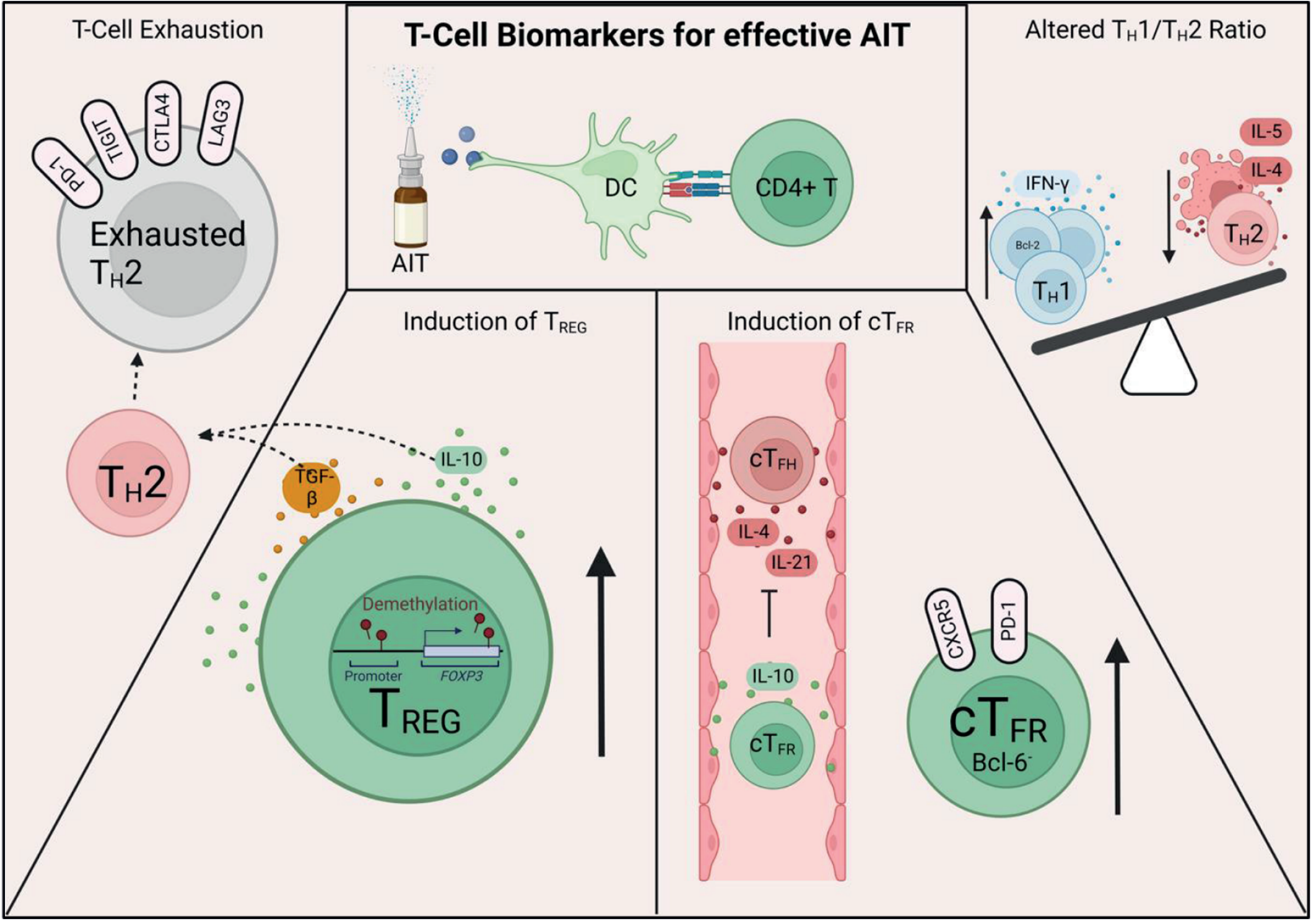
T-cell biomarkers in response to allergen immunotherapy. Chronic exposure to allergen results in a shift towards to T_H_1 immune state and deletion of T_H_2 cells, induction of regulatory T cells and T_FR_, and the exhaustion of effector T cells. Created with www.biorender.com.

Immune tolerance following SCIT and SLIT involves the deletion or anergy of effector cells, immune deviation to a T_H_1 response, and induction of T_REG_ cells (Figure 2). After 2 years of AIT, T_H_2-cell responses were inhibited and a lower level of IL4 mRNA was detected in the nasal mucosa following allergen challenge, in conjunction with a lower population of CRTH2^+^CCR4^+^CD27^-^CD161^+^ allergen-specific T_H_2A cells [16]. Higher levels of nasal IFN-γ resulting in a reduction of the IL-5/IFN-γ ratio upon allergen exposure was observed following SCIT [17]. Another study also demonstrated time course induction of IFN-γ, confirming the immune deviation towards T_H_1 responses [18]. T_H_1 responses are generally thought to be induced following apoptosis of T_H_2 cells. Cells obtained from AIT-treated grass pollen-allergic patients showed an increased number of IL-4^+^ cells undergoing apoptosis [19]. Additionally, T_H_1 cells were shown to have increased persistence through Bcl-2, an anti-apoptotic protein [20]. Therefore, a persistent T_H_1-cell population may be a biomarker of tolerance in AIT. 

T_FH_ cells expressing CXCR5 and PD-1 have been identified as mediators in inducing tolerance [21]. T_FH_ have now emerged to be an IL-4- and IL-21-producing subset [22]. An impaired T_FH_ and T_FR_ cell ratio was observed in patients with AR. T_FH_ cells in allergic patients have enhanced capacity to induce IgE production compared to healthy controls, whilst T_FR_ have diminished suppressive action [23]. Circulating T_FH_ (cT_FH_) cells were defined as a distinct subset of T cells from T_H_2 and T_H_2A cells lacking BCL-6 expression, efficient in secreting both IL-4 and IL-21 [24]. cT_FH_ cells were elevated in grass pollen-allergic patients compared to non-atopic controls and were lower following both SCIT and SLIT. In contrast, cT_FR_ and IL-10^+^ cT_FH_ cells were induced after SCIT and SLIT [24]. 

T_REGS_ are considered essential regulatory cells secreting IL-10 and TGF-β. Both natural (nT_REG_, FOXP3^+^) and induced (iT_REG_, CD49b^+^ IL-10^+^) T_REG_ cells have comparable suppressive function. They are characterized by low CD127 and high CD25 expression. Induction of T_REG_ subsets is commonly associated with immune tolerance after immunotherapy in allergic patients [25]. Multiple studies have shown correlation of T_REG_ frequency with SCIT and SLIT compared to a placebo [26, 27]. Increases in FOXP3 and IL-10 expression have been also observed. Additionally, CpG methylation within the FOXP3 regions following SLIT was reduced along with an increase in FOXP3 transcript expression [28]. Regulatory cytokines such as IL-10 and TGF-β promote T-cell exhaustion [29]. The proportion of PD-1^+^ CD4^+^ T cells was found to be increased following AIT treatment [30]. The transcriptional profile of CD4^+^ T cells following AIT demonstrated in another study showed that T_REG_ expressing c-MAF, NFIL3, LAG-3, TIGIT, PD-1, and TIM-3 were key markers of exhaustion [31]. Recent studies stimulating allergen specific CD4^+^ T cells showed a strong upregulation of PD-1, LAG-3, and CTLA4 upon in vitro stimulation with sensitizing allergen. The blocking of PD-1 re-invigorated T-cell activity by enhancing cytokine production in both allergic and non-allergic individuals [32]. AIT also resulted in the deletion of CD4^+^CD27^-^ T cells, where chronic exposure to high doses of allergens causes terminal differentiation of T_H_2 cells resulting in cell exhaustion [33]. Although these findings support T-cell exhaustion as a hallmark of long-term tolerance, more studies are warranted to identify their physiological role. T cells and other cellular biomarkers could be analyzed by isolation from whole blood samples. 

## Innate-cell biomarkers for effective treatment 

**Figure 3.**
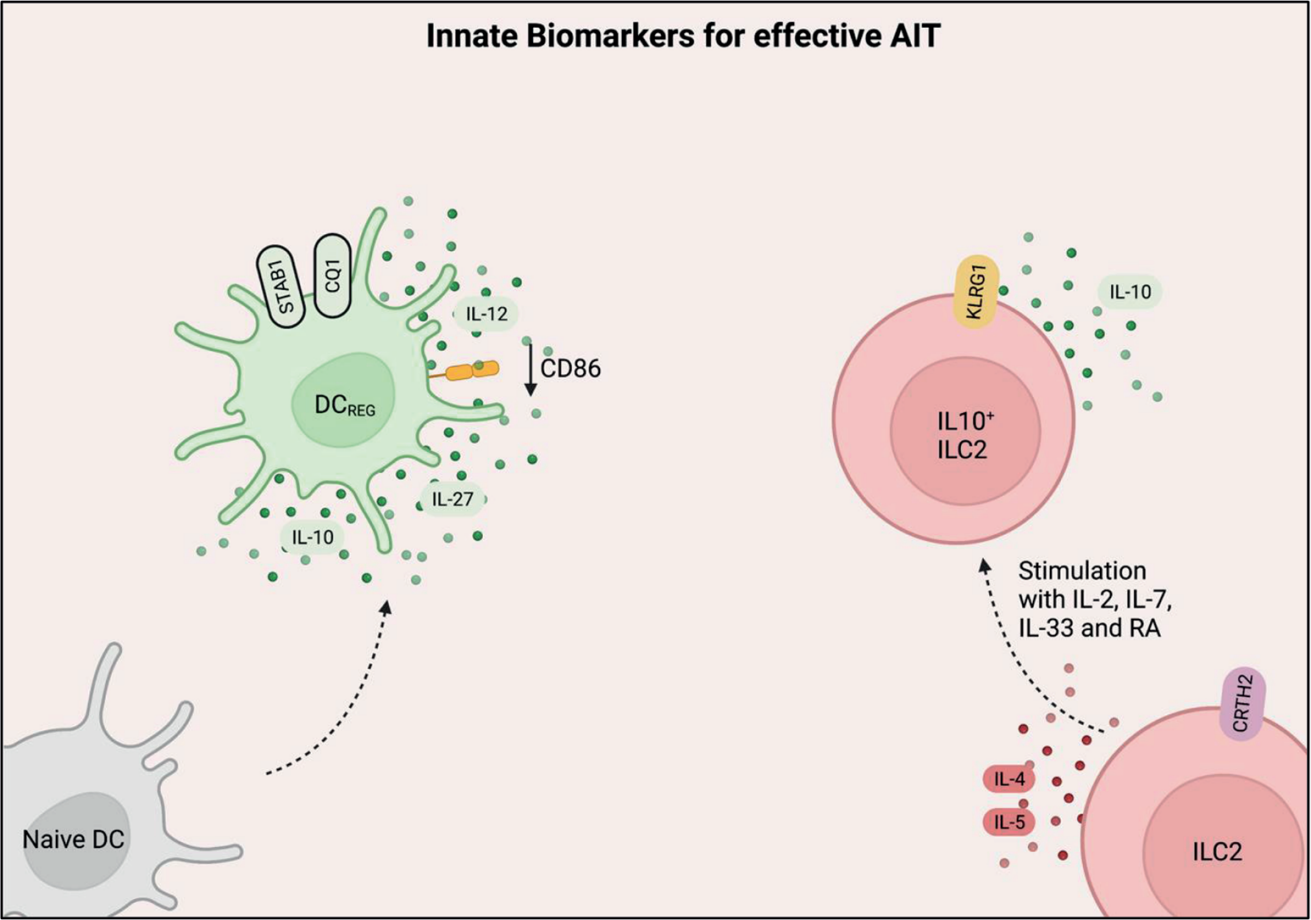
Innate responses towards effective allergen immunotherapy involve the induction of DC_REG_ and IL10-secreting ILC2s. Created with www.biorender.com.

Dendritic cells phagocyte allergen fragments and process them for MHC Class II presentation, resulting in induction of both inflammatory and regulatory cells. AIT induces regulatory DCs (DC_REG_), which have markers such as CQ1 and STAB1 [34, 35] (Figure 3). Moreover, DC_REG_ secrete IL-12, IL-27, and IL-10 whilst downregulating the expression of CD86, a co-stimulatory receptor that enhances inflammation [36]. ILC2s play key roles in SAR, and they mainly produce type 2 cytokines such as IL-5 and IL-13. SCIT treatment has been shown to lower the levels of ILC2 in both grass pollen and house dust mites (HDM) SCIT. Another novel ILC counterpart that produces IL-10 was also shown to be induced in AIT (Figure 3). Surprisingly, both SCIT and SLIT were shown to result in the induction of ILC2s that can produce IL-10 in vitro following stimulation of IL-2, IL-7, IL-33, and retinoic acid (RA) [37]. These observations show that ILCs play key roles in training immunity during AIT within the innate compartment. 

## B-cell biomarkers indicating effective AIT 

**Figure 4.**
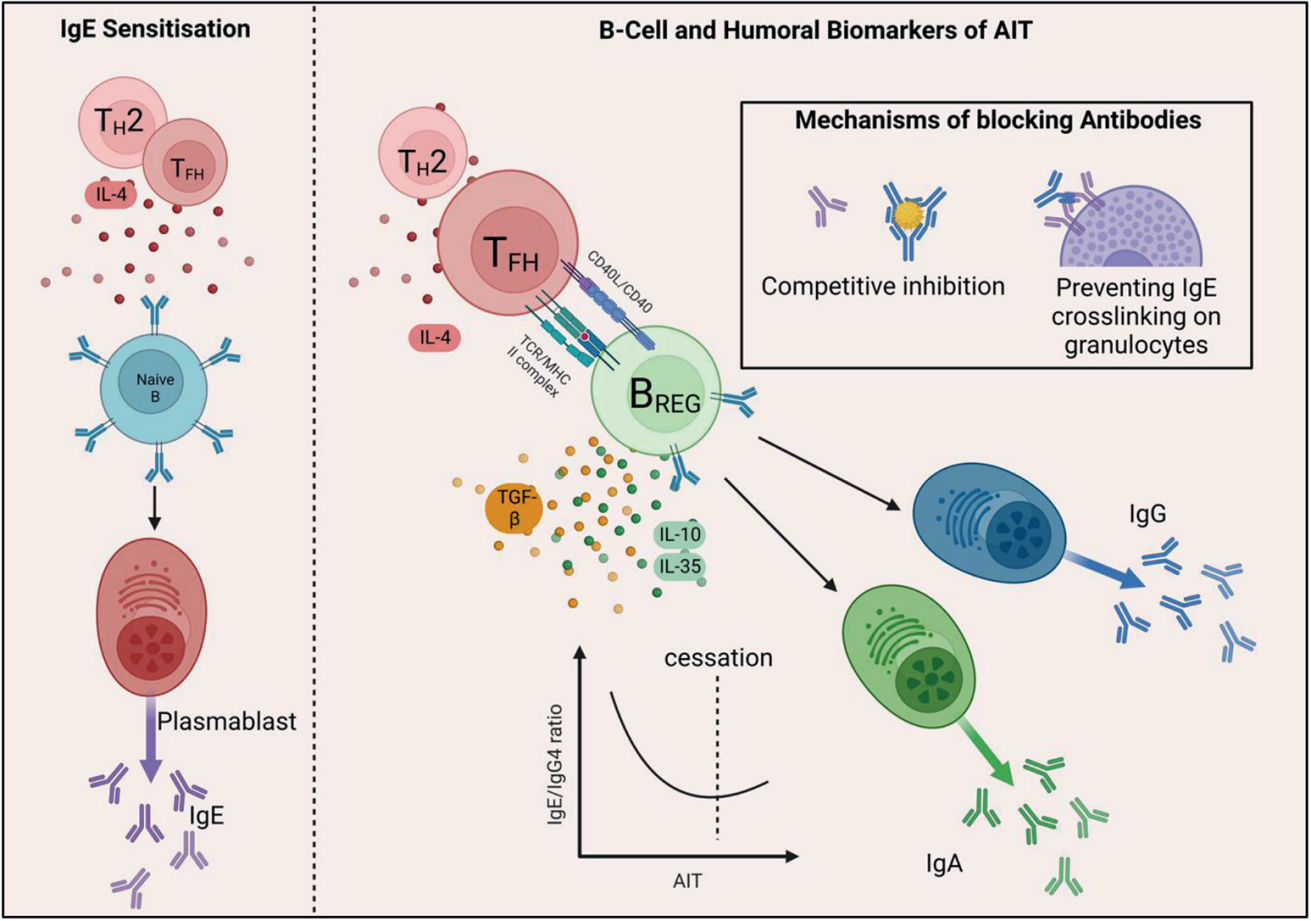
B-cell and humoral responses to allergen immunotherapy. Chronic allergen exposure results in the induction of B_REG_ that secret IL-10, IL-35, and TGF-β. The development of B cells producing blocking antibodies IgA and IgG are also positive indicators of successful therapy. Created with www.biorender.com.

B_REGS_ exert negative immunoregulatory roles through the production of different cytokines such as IL-10, IL-35, TGF-β, and through cell contact mechanisms [38] (Figure 4). B-cell activation is required for suppressive action by the activation of toll-like receptors, BCR signaling, and co-stimulation via CD40/CD40L and CD80/CD86. Inflammatory cytokines also activate B_REGS_ via STAT3. Grass pollen and HDM AIT result in elevated levels of IgA- and IgG4-expressing B cells, plasmablasts, and IL-10^+^ B_REGS_, which correlated with improvement of clinical symptoms throughout AIT [39]. 

## Ig responses indicative of effective AIT 

AIT success requires the immunomodulation of both cellular and humoral responses. In AIT, the induction of allergen-specific IgG, IgG4, and IgA, which have IgE-blocking activity, has been well characterized [40], and their upregulation seems to be in line with clinical symptoms [41]. The main mechanism involves competitively inhibiting IgE for allergen binding, resulting in the reduction of IgE-induced FcεRI activation on mast cells, thus reducing degranulation and release of inflammatory mediators [42]. Additionally, binding of allergen-IgE complexes to low-affinity IgE receptors on B cells is inhibited, resulting in diminished IgE-facilitated presentation to T cells [40] (Figure 4). The IgE-FAB assay showed that blocking antibody activity following SCIT was time- and dose-dependent, peaking between 3 and 6 months [43]. The GRASS trial revealed that SCIT or SLIT is associated with IgG- or IgA-blocking antibodies, respectively [15]. Humoral biomarkers could be measured both systemically or locally in serum and nasal fluids; and they could be useful to predict local responses characteristic of early AIT response [42]. 

## Epigenetics and metabolomics as indicators of effective AIT 

Omics sciences have been recently used in an attempt to find biomarkers that could be indicative of an effective response to AIT, and to better determine the underlying mechanisms that lead to AIT success, such as the desensitization of effector cells or the shifting towards a regulatory immune response. This high-throughput approach of working with large datasets with genomics, microbiomics, transcriptomics, proteomics, and metabolomics could significantly facilitate the discover of new biomarkers [44]. Moreover, omics biomarkers can be measured in a wide range of matrices, including serum, nasal fluid, or exhaled breath condensates [45]. Thanks to omics, key factors to a successful AIT treatment, like an appropriate characterization of allergen extracts or a deep profiling of the patients’ IgE reactivity have been discovered [46, 47, 48]. AIT has shown to induce several epigenetic changes in T cells, such as the induction of FOXP3 [28]; and also in monocytes and DCs, including the downregulation of *EZH2* gene in DCs [49] and a tolerogenic reprogramming of monocyte and DCs [50]. 

**Figure 5.**
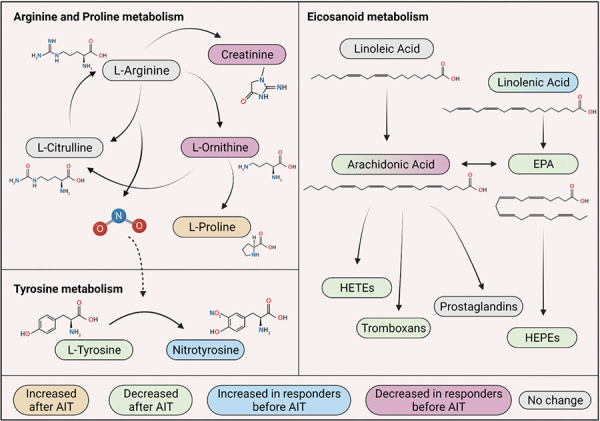
Pathways that have been found altered in allergen immunotherapy. Nitric Oxid-related metabolites, along with eicosanoid metabolism, could serve as biomarkers to find responder patients or assess the success of the treatment. Created with www.biorender.com.

Regarding metabolomics, there are currently several proposed biomarkers that could predict response to AIT. It has been recently suggested that the sensitization of the patient (either to one or multiple allergens) is relevant to predict their response to AIT [51]. Moreover, a decrease in eicosanoids has been reported after treatment with SCIT for allergy and allergic asthma [52, 53]. Eicosanoids are known to enhance inflammation, with its precursor, the arachidonic acid pathway being increased in severe, uncontrolled allergic asthma [54]. One such evidence includes a study by Xie et al. [55] which demonstrated twelve biomarkers that were found to be different between responsive and unresponsive patients prior to the treatment with SLIT. These included arachidonic acid, sphingosine, or L-phenylalanine, all of which correlated with altered energetic and inflammatory pathways and the arginine and proline metabolism [55]. L-tyrosine was also found to have an opposite trend between patients with a good and a bad response to SCIT treatment (being decreased after successful AIT and increased for patients for whom it was ineffective); and nitric oxide related pathways (such as arginine and proline metabolism, tyrosine metabolism and nitrogen metabolism) were altered during AIT [56] (Figure 5). 

## Conclusion 

Mechanistic studies have allowed a more holistic understanding of the underlying pathways induced by AIT. The efficacy is associated with the inhibition of inflammatory responses and the induction of markers in both adaptive and innate immune systems. Additional studies are required to elucidate the molecular and epigenetic changes and differences in different modes of AIT. Ultimately, a panomic approach is required to uncover full mechanisms which may prove instrumental to develop new targeted therapies. 

## Funding 

None. 

## Conflict of interest 

M.H. Shamji reports grants from Regeneron, Merck, ANGANY Inc, Allergy Therapeutics, and Immune Tolerance Network; reports personal fees from Allergopharma; and reports grants and personal fees from ALK, Allergy Therapeutics, and ANGANY Inc.
